# Multicentre validation of CT grey-level co-occurrence matrix features for overall survival in primary oesophageal adenocarcinoma

**DOI:** 10.1007/s00330-024-10666-y

**Published:** 2024-03-25

**Authors:** Robert O’Shea, Samuel J. Withey, Kasia Owczarczyk, Christopher Rookyard, James Gossage, Edmund Godfrey, Craig Jobling, Simon L. Parsons, Richard J. E. Skipworth, Vicky Goh, on behalf of the OCCAMS Consortium, Rebecca C. Fitzgerald, Paul A. W. Edwards, Nicola Grehan, Barbara Nutzinger, Aisling M. Redmond, Sujath Abbas, Adam Freeman, Elizabeth C. Smyth, Maria O’Donovan, Ahmad Miremadi, Shalini Malhotra, Monika Tripathi, Calvin Cheah, Hannah Coles, Matthew Eldridge, Maria Secrier, Ginny Devonshire, Sriganesh Jammula, Jim Davies, Charles Crichton, Nick Carroll, Richard H. Hardwick, Peter Safranek, Andrew Hindmarsh, Vijayendran Sujendran, Stephen J. Hayes, Yeng Ang, Andrew Sharrocks, Shaun R. Preston, Izhar Bagwan, Vicki Save, J. Robert O’Neill, Olga Tucker, Andrew Beggs, Philippe Taniere, Sonia Puig, Gianmarco Contino, Timothy J. Underwood, Ben L. Grace, Jesper Lagergren, Andrew Davies, Fuju Chang, Ula Mahadeva, Francesca D. Ciccarelli, Grant Sanders, David Chan, Ed Cheong, Bhaskar Kumar, Loveena Sreedharan, Irshad Soomro, Philip Kaye, John Saunders, Laurence Lovat, Rehan Haidry, Michael Scott, Sharmila Sothi, George B. Hanna, Christopher J. Peters, Krishna Moorthy, Anna Grabowska, Richard Turkington, Damian McManus, Helen Coleman, Russell D. Petty, Freddie Bartlett, Tom D. L. Crosby

**Affiliations:** 1 Department of Cancer Imaging, School of Biomedical Engineering and Imaging Sciences, King’s College London, London, UK; 2 Department of Radiology, Royal Marsden Hospital NHS Trust, Sutton, Surrey UK; 3 Department of Clinical Oncology, Guy’s & St Thomas’ Hospitals NHS Foundation Trust, London, UK; 4 Department of Surgery, Guy’s & St Thomas’ Hospitals NHS Foundation Trust, London, UK; 5 Department of Radiology, Cambridge University Hospitals NHS Foundation Trust, Cambridge, UK; 6 Department of Radiology, Nottingham University Hospitals NHS Foundation Trust, Nottingham, UK; 7 Department of Surgery, Nottingham University Hospitals NHS Foundation Trust, Nottingham, UK; 8 Department of Surgery, Edinburgh Royal Infirmary, NHS Lothian, Edinburgh, UK; 9 Department of Radiology, Guy’s & St Thomas’ Hospitals NHS Foundation Trust, Westminster Bridge Road, London, SE1 7EG UK

**Keywords:** Oesophageal neoplasms, Adenocarcinoma, Radiomics, Prognosis, Precision medicine

## Abstract

**Background:**

Personalising management of primary oesophageal adenocarcinoma requires better risk stratification. Lack of independent validation of proposed imaging biomarkers has hampered clinical translation. We aimed to prospectively validate previously identified prognostic grey-level co-occurrence matrix (GLCM) CT features for 3-year overall survival.

**Methods:**

Following ethical approval, clinical and contrast-enhanced CT data were acquired from participants from five institutions. Data from three institutions were used for training and two for testing. Survival classifiers were modelled on prespecified variables (‘Clinical’ model: age, clinical T-stage, clinical N-stage; ‘ClinVol’ model: clinical features + CT tumour volume; ‘ClinRad’ model: ClinVol features + GLCM_Correlation and GLCM_Contrast). To reflect current clinical practice, baseline stage was also modelled as a univariate predictor (‘Stage’). Discrimination was assessed by area under the receiver operating curve (AUC) analysis; calibration by Brier scores; and clinical relevance by thresholding risk scores to achieve 90% sensitivity for 3-year mortality.

**Results:**

A total of 162 participants were included (144 male; median 67 years [IQR 59, 72]; training, 95 participants; testing, 67 participants). Median survival was 998 days [IQR 486, 1594]. The ClinRad model yielded the greatest test discrimination (AUC, 0.68 [95% CI 0.54, 0.81]) that outperformed Stage (ΔAUC, 0.12 [95% CI 0.01, 0.23]; *p* = .04). The Clinical and ClinVol models yielded comparable test discrimination (AUC, 0.66 [95% CI 0.51, 0.80] vs. 0.65 [95% CI 0.50, 0.79]; *p* > .05). Test sensitivity of 90% was achieved by ClinRad and Stage models only.

**Conclusions:**

Compared to Stage, multivariable models of prespecified clinical and radiomic variables yielded improved prediction of 3-year overall survival.

**Clinical relevance statement:**

Previously identified radiomic features are prognostic but may not substantially improve risk stratification on their own.

**Key Points:**

*• Better risk stratification is needed in primary oesophageal cancer to personalise management.*

*• Previously identified CT features—GLCM_Correlation and GLCM_Contrast—contain incremental prognostic information to age and clinical stage.*

*• Compared to staging, multivariable clinicoradiomic models improve discrimination of 3-year overall survival.*

**Supplementary information:**

The online version contains supplementary material available at 10.1007/s00330-024-10666-y.

## Introduction

Oesophageal cancer presents a major burden worldwide [1]. A multimodal treatment approach (surgery with chemotherapy or chemoradiotherapy) is standard of care following landmark trials [2–4] and offers the best chance of survival for resectable cancer. However, despite this, outcome remains poor for patients treated with curative intent with mortality rates of 45–53% in the first year post diagnosis [5] and median post-progression survival of only 13 months [6]. There is a greater need for personalisation of management to obviate treatment in patients who may not benefit substantially. Alongside this, with the growing interest in total neoadjuvant therapy, and ongoing trials of perioperative immunotherapy, better initial risk stratification of patients at diagnosis is needed to guide management.

Currently, the clinical TNM (tumour-node-metastasis) stage guides management [7] but it has a low predictive accuracy with contemporaneous pathological stage for early stage cancers [8], as well as limitations in prognostication. Prognostic information has central importance for patient decision-making, with cancer patients ranking prognosis, diagnosis and treatment options as their highest information priority [9].

Modelling studies have demonstrated scope to improve upon prognostication in oesophageal adenocarcinoma, including through CT imaging radiomic approaches [10–12]. Initial publications have highlighted the potential of different locoregional features. For example, Piazzese et al identified an association of CT grey-level zone distance variance with overall survival in a multicentre cohort (*n* = 213), which was independent of dimensionality and contrast administration [11], whilst Larue et al developed a random forest radiomic model including 40 CT features to predict 3-year overall survival (OS) in oesophageal cancer patients treated with chemoradiotherapy (*n* = 239) [12].

To date, clinical translation of imaging radiomic models has been hampered by a relative paucity of independent external validation. Thus, we aimed to validate a prognostic model for 3-year OS including prespecified clinical features and previously proposed CT radiomic features in a prospective multicentre setting for patients with primary oesophageal adenocarcinoma planned for curative treatment.

## Methods

### Participants and datasets

Following ethical approval, clinical data and CT imaging were obtained prospectively from five institutions participating in the OCCAMS (Oesophageal Cancer Clinical and Molecular Stratification) Consortium. Consecutive participants with non-metastatic, pathologically proven oesophageal adenocarcinoma who underwent staging contrast-enhanced CT imaging and planned for definitive treatment were eligible. Participants were excluded if (1) no tumour was visible on CT; (2) CT images were unavailable/corrupted; and (3) concurrent malignancy was present. Data from three institutions were used for model development and two institutions for model testing.

### CT imaging and analysis

Contrast-enhanced CT was performed according to institutional practice and included arterial phase imaging of the thorax and upper abdomen. CT acquisition and reconstruction parameters for the training and test datasets are summarised in Supplementary Table 1. Pre-processing of CT images was undertaken as per Image Biomarker Standardisation Initiative recommendations [13]. CT slice thickness was linearly interpolated to 2 mm and attenuation values were converted to Hounsfield units with PyDICOM [14]. The primary tumour was segmented by a radiologist (with 5 years’ experience), who was blinded to clinical outcomes, on the arterial phase thoraco-abdominal CT images. The rationale for using the arterial versus portal venous phase was prior studies showing better tumour conspicuity [15] and tumour staging accuracy [16] for the arterial phase. Tumour segmentations were reviewed and adjusted as required by a second radiologist (with > 20 years’ experience). Examples of tumour segmentations are shown in Supplementary Figs. 1 and 2. 3D Radiomic features were extracted from the segmented volume of interest using PyRadiomics version 3.0.1 under default parameters (no image filters, no normalisation, no voxel array shift, grey-level discretisation at fixed bin width of 25 Hounsfield units) [17].

### Radiomic feature selection

Following recommendations [18], radiomic features were pre-selected based on previous published studies. This obviated data-driven feature selection thereby reducing the risk of data overfitting. Published studies were evaluated using the ‘Transparent reporting of a multivariable prediction model for individual prognosis’ (TRIPOD) [19] and ‘Radiomics Quality Score’ checklists [20]. Identifiable Image Biomarker Standardisation Initiative features were sought [13].

Of the identified published studies [11, 12, 21, 22], features proposed by Piazzese et al were excluded as the interpolation strategy employed (2 mm isotropic) would have resulted in a significant reduction of the axial image resolution [11]. Features proposed by Larue et al were excluded as they evaluated nonlinear effects in a high-dimensional feature set using a random forest method, complicating the extraction of a few individually informative linear predictions [12]. Zhang et al achieved a high TRIPOD score of 30 [21] and examined a limited number of previously proposed predictors, each of which was identifiable; thus, these features were extracted—GLCM_Contrast, GLCM_Correlation and GLCM_InverseDifferenceMoment. Furthermore, GLCM correlation was independently identified as a predictor of response in Klaasen et al [22]. Following Peduzzi and Concato’s guideline recommendation of more than 10 events per modelled feature [23], an unsupervised method was applied to identify the most suitable two of the three proposed features. The most collinear GLCM feature with respect to tumour volume and the other GLCM features was excluded.

### Statistical analysis

Differences of participant characteristics between training and testing datasets were tested with the Fisher test for categorical variables or the two-sided *t*-test for continuous variables. For modelling, unpenalised logistic regression models were fitted to predict 3-year OS using base R. With a median survival of 24 months following surgery alone and 46 months following neoadjuvant chemoradiotherapy and surgery, 3-year OS is a meaningful endpoint in oesophageal cancer and has been used in clinical trials assessing the efficacy of neoadjuvant treatment [24].

The following four models were fitted, using the following sets of features:

1. ‘Stage’: overall TNM stage.
2. ‘Clinical’: age, clinical T-stage and N-stage, as determined at tumour board review
3. ‘ClinVol’: age, clinical T-stage and N-stage, primary CT tumour volume
4. ‘ClinRad’: age, clinical T-stage and N-stage, primary tumour volume, two GLCM features.

Discrimination and calibration of 3-year OS were assessed using the area under the receiver operator curve (AUC) and Brier score, respectively, using the riskRegression R library [25]. Confidence intervals were estimated using the method of Blanche [26] and compared using the Delong test. Following Van Rossum [27], clinical utility was assessed by thresholding the model prediction in training data to maximise specificity whilst maintaining sensitivity of > 90%. Confidence intervals for sensitivity, specificity and accuracy were estimated with 1000 replacement bootstraps.

To ensure absence of dataset-partitioning bias or institutional confounding, supplementary post hoc model evaluation was performed using each institution in turn for testing and the remaining four institutions for model fitting. Kaplan–Meier curves were plotted, grouping participants according to the target 90% sensitivity threshold fitted in training data.

A post hoc analysis was also performed to estimate conditional dependencies between radiomic features and survival time, using data from training and testing cohorts. A partial Spearman correlation matrix was inferred using the de-sparsified graphical least absolute shrinkage and selection operator method via the SILGGM package and 95% confidence intervals were estimated via bootstrapping with 1000 replicates. Spearman correlation was also employed to assess volume confounding of radiomic features. Analysis of variance was employed to test radiomic stability with respect to scanner manufacturer and study institution.

## Results

### Participant and dataset characteristics

**Fig. 1:**
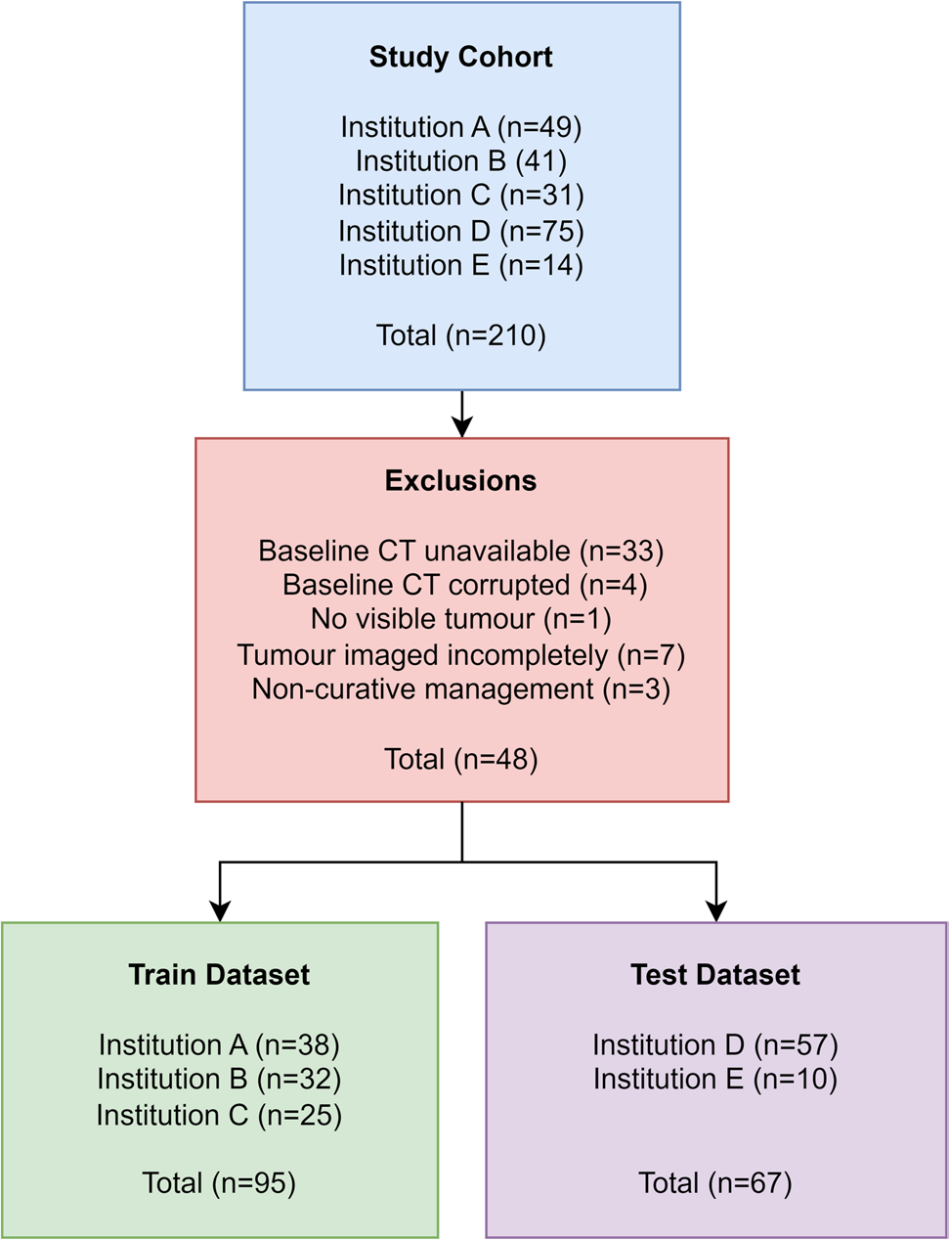
Study participant flowchart

Of 210 participants recruited, 48 participants were excluded, generating a training set of 95 participants and a test set of 67 participants. The participant flowchart is provided in Fig. 1 showing the reasons for exclusions. Participant and dataset characteristics are shown in Table 1 and Supplementary Fig. 3.

**Table 1:** Participant characteristics in training and testing datasets

Variable	Value	Training	Test	*p*-value
Age *(mean, SD)*	Years	65 ± 9	65 ± 9	.89
Sex *(n, %)*	Male	11 (12)	7 (10)	
	Female	84 (88)	60 (90)	.99
Clinical T-stage *(n, %)*	T1	1 (1)	0 (0)	.45
	T2	16 (17)	11 (16)	
	T3	77 (81)	53 (79)	
	T4	1 (1)	3 (4)	
Clinical N-stage *(n, %)*	N0	24 (25)	13 (19)	.12
	N1	36 (38)	38 (57)	
	N2	32 (34)	14 (21)	
	N3	3 (3)	2 (3)	
Location *(n, %)*	Oesophagus, mid	5 (5)	0 (0)	< .001
	Oesophagus, lower	51 (54)	12 (18)	
	Gastrooesophageal junction	39 (41)	55 (82)	
Treatment *(n, %)*	Chemotherapy +/- radiotherapy and surgery	81 (85)	65 (97)	.005
	Chemoradiotherapy only	1 (1)	2 (3)	
	Surgery only	13 (14)	0 (0)	
Survival status at 3 years *(n, %)*	Deceased	57 (60)	30 (45)	.08
	Survived	38 (40)	37 (55)	

### Performance of model variables

**Fig. 2:**
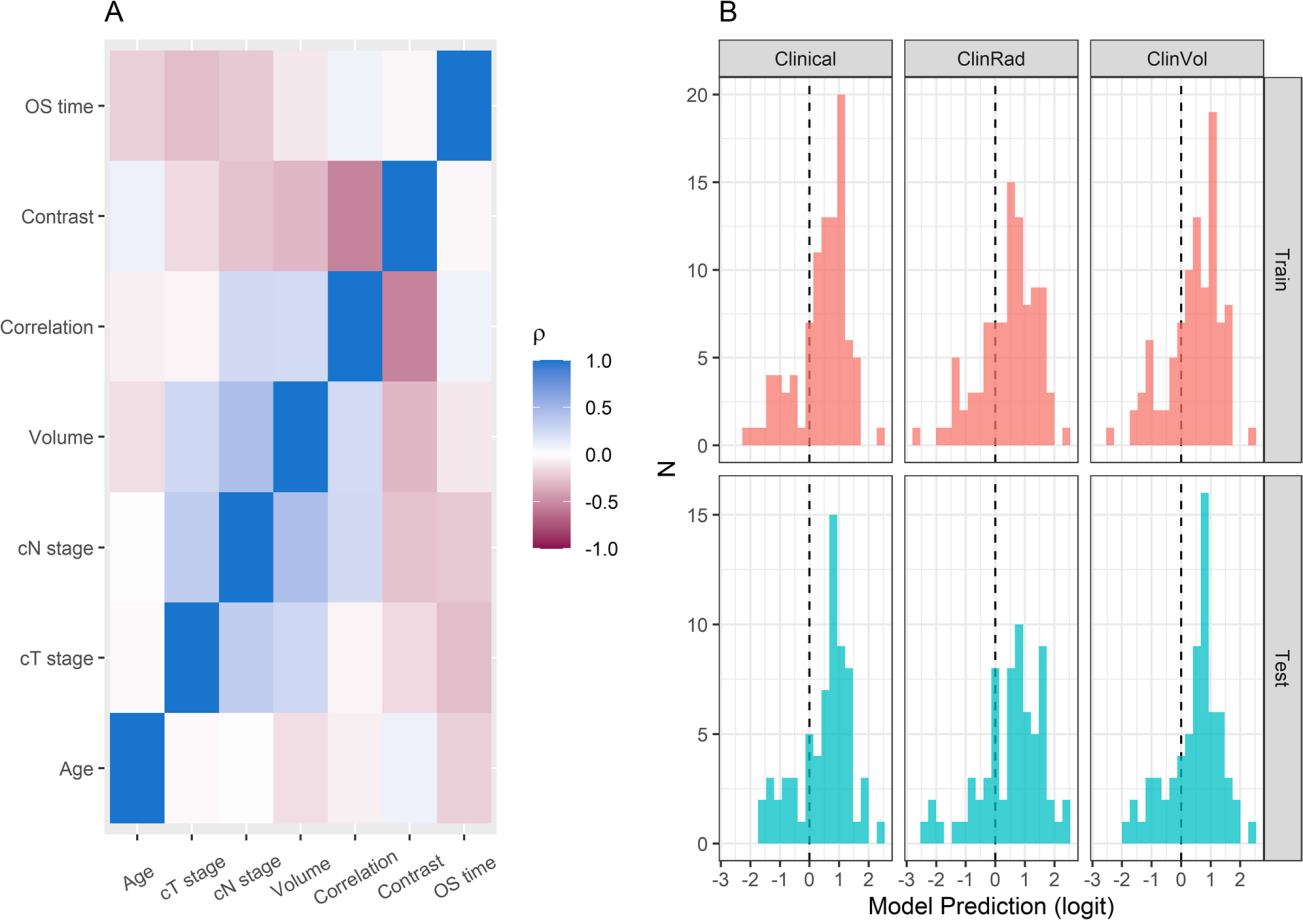
Spearman correlations of predictor variables in training data (**A**) and histograms of model predictions in training and testing data (**B**) are shown

**Fig. 3:**
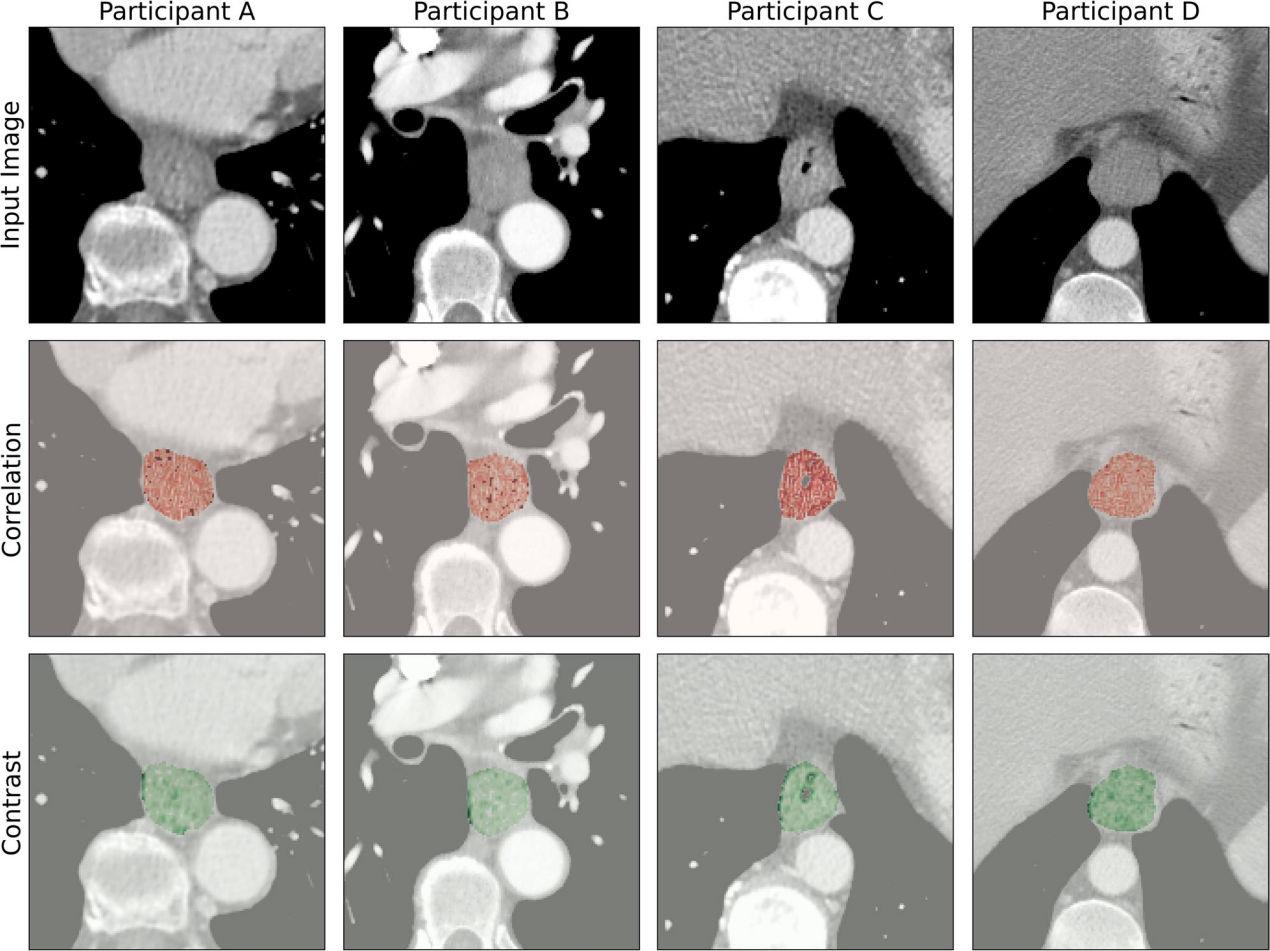
Visualisation of CT images and voxel-level radiomic features (GLCM correlation and GLCM contrast) in four study participants. Participant **A**: 71-year-old female with a clinically staged T2 tumour [correlation 0.67, contrast 1.22]; participant **B**: 78-year-old female with a clinically staged T3 tumour [correlation 0.5, contrast 2.56]; participant **C**: 62-year-old male with a clinically staged T2 tumour [correlation 0.48, contrast 3.01]; participant **D**: 56-year-old male with a clinically staged T3 tumour [correlation 0.57, contrast 2.10]

Spearman correlations of the prespecified predictors in the training data are shown in Fig. 2A. GLCM_InverseDifferenceMoment was highly correlated with tumour volume (*r* =  + 0.33) and GLCM_Contrast (*r* =  − 0.94). Hence, GLCM_Correlation and GLCM_Contrast were selected for modelling (Supplementary Material). Clinical T-stage was the most important prognosticator, with each model assigning it a significant positive coefficient (representing increasing risk with increasing stage). The second most influential predictor was age, which was assigned significant positive coefficients in each model. Tumour volume was an insignificant predictor in both the ClinVol and ClinRad models. GLCM_Correlation was assigned a marginal negative coefficient in the ClinRad model, and GLCM_Contrast was the least influential model predictor. Model coefficients, their standard errors and associated *z*-tests are reported in Table 2. Histograms of model predictions demonstrated that each model had similar distributions of predictions in training and testing (Fig. 2B). Radiomic features are visualised at voxel level in Fig. 3.

**Table 2:** Summary of model coefficients

Model	Variable	Coefficient	Standard error	*p*-value
Clinical	(Intercept)	− 7.49	2.57	.004
	Age	0.05	0.03	.05
	cT-stage	1.45	0.61	.02
	cN-stage	0.32	0.30	.28
ClinVol	(Intercept)	− 5.75	4.09	.16
	Age	0.05	0.03	.06
	cT-stage	1.50	0.62	.02
	cN-stage	0.39	0.32	.23
	Volume (log cm^3)	− 0.18	0.33	.59
ClinRad	(Intercept)	− 5.16	4.43	.24
	Age	0.05	0.03	.05
	cT-stage	1.41	0.64	.03
	cN-stage	0.48	0.33	.14
	Volume (log cm^3)	− 0.10	0.34	.76
	GLCM_Correlation	− 2.45	2.20	.26
	GLCM_Contrast	− 0.03	0.32	.92

Logistic regression coefficients and *p-*values from unadjusted two-tailed *z*-tests are reported

### Prediction of 3-year overall survival

The ClinRad model showed best discrimination of 3-year OS, achieving similar performance in both training (AUC, 0.71 [95% CI 0.60, 0.82]) and testing (AUC, 0.68 [95% CI 0.54, 0.81]) (Table 3). Test discrimination of the ClinRad model was greater than that of Stage alone (Δ AUC, 0.12 [95% CI 0.01, 0.23]; *p* = 0.04). Stage was the least discriminative model in both training (AUC, 0.60 [95% CI 0.49, 0.71]) and testing (AUC, 0.56 [95% CI 0.44, 0.67]).

**Table 3:** Model discrimination and calibration metrics with respect to 3-year overall survival

Dataset	Model	AUC	Brier score	Sensitivity*	Specificity*	Accuracy*
Train	Stage	0.60 [0.49, 0.71]	0.24 [0.21, 0.28]	0.95 [0.89, 1.00]	0.21 [0.09, 0.35]	0.65 [0.56, 0.74]
Train	Clinical	0.69 [0.57, 0.80]	0.22 [0.18, 0.27]	0.91 [0.83, 0.98]	0.35 [0.21, 0.50]	0.69 [0.60, 0.78]
Train	ClinVol	0.70 [0.58, 0.81]	0.22 [0.18, 0.27]	0.91 [0.84, 0.98]	0.34 [0.20, 0.49]	0.68 [0.58, 0.77]
Train	ClinRad	0.71 [0.60, 0.82]	0.22 [0.17, 0.26]	0.93 [0.86, 0.98]	0.29 [0.14, 0.44]	0.67 [0.58, 0.77]
Test	Stage	0.56 [0.44, 0.67]	0.27 [0.24, 0.31]	0.93 [0.83, 1.00]	0.14 [0.03, 0.26]	0.49 [0.37, 0.61]
Test	Clinical	0.66 [0.51, 0.80]	0.28 [0.23, 0.33]	0.83 [0.68, 0.96]	0.22 [0.09, 0.36]	0.49 [0.37, 0.61]
Test	ClinVol	0.65 [0.50, 0.79]	0.28 [0.23, 0.33]	0.83 [0.69, 0.96]	0.19 [0.07, 0.32]	0.48 [0.36, 0.60]
Test	ClinRad	0.68 [0.54, 0.81]	0.28 [0.22, 0.33]	0.90 [0.77, 1.00]	0.19 [0.06, 0.33]	0.51 [0.39, 0.63]

*Sensitivity, specificity and accuracy were measured at a risk threshold which achieved at least 90% sensitivity in training data. 95% confidence intervals are provided in square brackets

The Clinical model achieved similar test discrimination to the ClinRad model (AUC, 0.66 [95% CI 0.51, 0.80]; Δ AUC, 0.02 [95% CI − 0.04, 0.08]; *p* > 0.05). The ClinVol model attained marginally lower test discrimination than the Clinical model (AUC, 0.65 [95% CI 0.50, 0.79]). All models yielded similar test calibration. Only Stage and ClinRad models achieved target 90% sensitivity in both training and testing. Here, the ClinRad model yielded slightly higher specificity (specificity, 0.19 [95% CI 0.06, 0.33]) than Stage (specificity, 0.14 [95% CI 0.03, 0.26]) at this threshold.

**Fig. 4:**
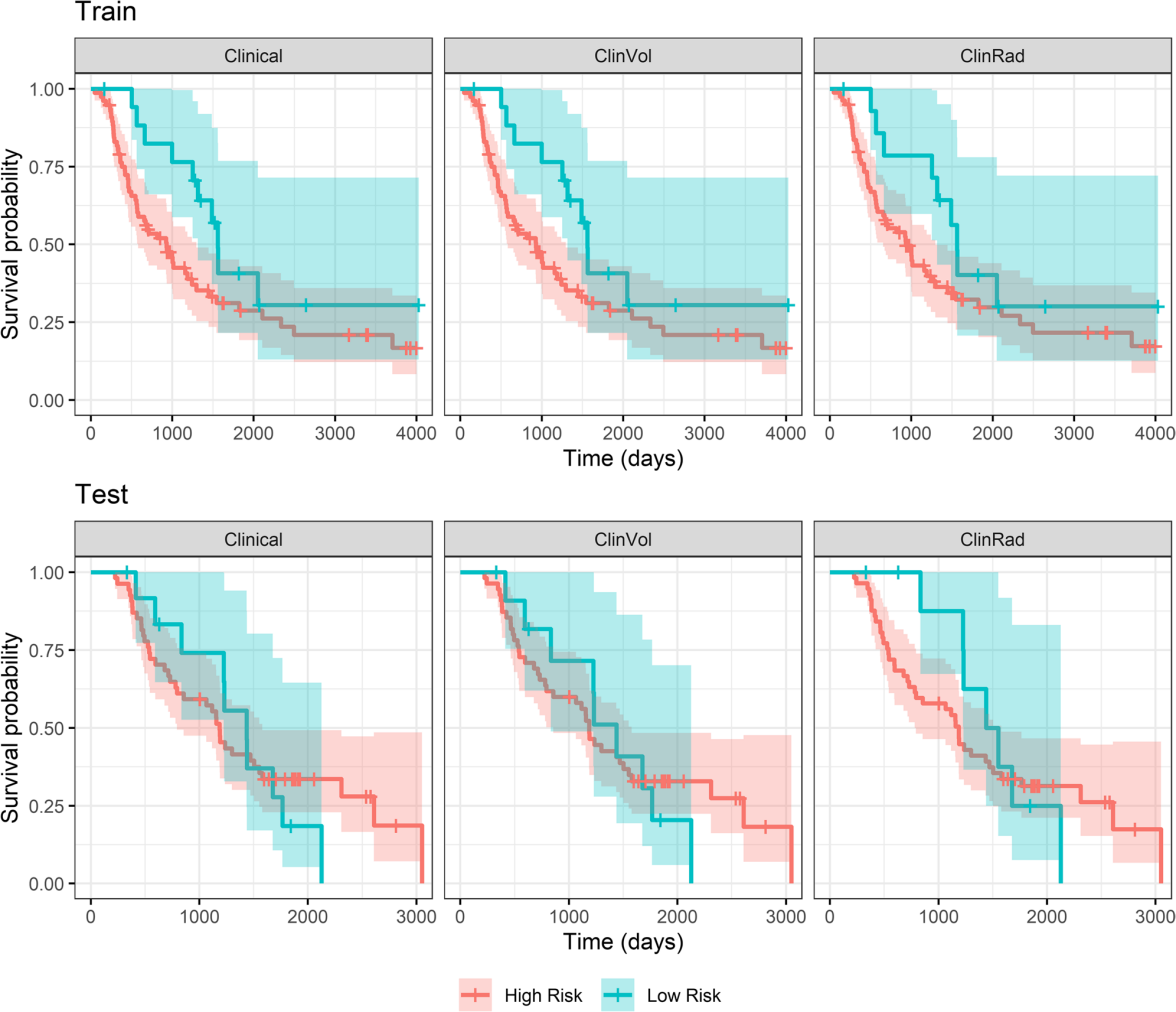
Kaplan–Meier plot of survival in high- and low-risk groups according to each model score. Risk groups were defined according to the target 90% sensitivity threshold fitted in training data

Supplementary per-institution model testing results were consistent with the main external validation results (Supplementary Table 2). Kaplan–Meier curves are provided in Fig. 4. Survival statistics are provided in Supplementary Table 3. In the test data, risk groups assigned by the ClinRad model separated survival curves for the initial 3 years, and convergence was observed at 5 years. However, few participants were assigned to the high-risk group (9/67, 13%). Risk groupings assigned by Clinical and ClinVol models achieved little separation of survival curves in testing. Post hoc partial correlation analysis confirmed that, over both training and validation datasets, the most informative predictors of overall survival time were clinical N-stage (partial *ρ*, −0.15 [95% CI − 0.33, 0.02]) and age (partial *ρ*, − 0.14 [95% CI − 0.3, 0.02]). The level of independent predictive information contributed by GLCM_Correlation (partial *ρ*, 0.10 [95% CI − 0.05, 0.25]) was comparable to that of clinical T-stage (partial *ρ*, − 0.12 [95% CI − 0.26, 0.04]). Partial correlation analysis results are provided in Supplementary Table 4. Feature variability with respect to scanner manufacturer and study institution are also presented in Supplementary Material.

## Discussion

The ability to provide individualised risk–benefit analysis would help to optimise management decisions in potentially resectable oesophageal cancer. Improving prognostication is a step in this direction as prognosis influences treatment decisions made by doctors and patients. Surgery may have an impact on quality of life for up to 12 months post treatment [28] and patients with a poorer prognosis may not fully benefit from a multimodal approach. Initial imaging studies have suggested that radiomic features may have additive prognostic value [11, 12, 18, 21]. However, prespecified models have to demonstrate reliable prediction in external datasets without local refitting. Accordingly, studies need to transition to the evaluation of previously proposed predictors and models, rather than continuing to fit new models with many degrees of freedom to new clinical data [18].

In this prospective multicentre study, we have demonstrated that a multivariate clinicoradiomic prognostic model (ClinRad) incorporating previously identified CT features improved discrimination of 3-year OS compared to TNM staging with an AUC of 0.68 in the test dataset, but offered similar calibration. The Clinical model had similar performance as the ClinRad model with an AUC of 0.66. Both Clinical and ClinRad models retained discriminative capacity between training and testing, though calibration deteriorated, suggesting a distributional mean shift between institutions.

Our findings are concordant with previously published data of Larue et al [12], where the high-dimensional random forest radiomic model with other features achieved AUCs of 0.69 and 0.61 in training and testing, respectively. The direction of radiomic coefficients fitted in this study is consistent with previously published results by Zhang et al [21], who observed increasing GLCM_Correlation in patients with oesophageal adenocarcinoma who responded to chemoradiotherapy. In our model, low GLCM_Correlation was an adverse prognosticator. Our finding that GLCM correlation was the most informative predictor also concurs with Klaasen et al [22]. However, as Klaasen’s model employed a random forest architecture, the directional concordance of results could not be verified.

Zhang et al [21] also observed decreasing GLCM contrast in chemoradiotherapy responders. In our study, GLCM contrast did not affect model predictions substantially, indicating that any prognostic information it encoded was already provided by the other clinical and image-based predictors already modelled.

An advantage of our study is that it incorporated multicentre prospective data, thereby providing realistic conditions for the estimation of model informativeness and generalisability. The imaging equipment and protocols were representative of the varying conditions encountered in clinical practice. The imaging acquisition parameters in this dataset reflected typical clinical practice and variations between institutions, which a radiomic model must be able to accommodate. We noted that GLCM correlation and GLCM contrast varied according to institution and scanner manufacturer respectively. This variability introduces noise which can complicate modelling of the underlying prognostic signal. Clinical deployment of radiomic models requires either that this noise is accommodated or that clinical imaging protocols adapt to acquire images under more standard conditions.

Model validation was performed in test data from three institutions which were unobserved during model development, yielding a realistic estimate of model generalisability in our healthcare system. However, our study had limitations. First, manual segmentation especially of early-stage cancers is subject to intra-reader and inter-reader variability [29]. Second, radiomic approaches are not typically well suited for the identification of new imaging biomarkers, due to the low ratio of events to evaluated variables [18, 20]. It is noteworthy that the ClinRad model fitted here is simpler than that of Larue, whilst matching its training performance, and marginally improving upon its generalisation [12]. However, a necessary cost of this study design is that the other informative radiomic features may have been omitted. Third, although both the ClinRad model and TNM staging demonstrated 90% sensitivity, the low specificity achieved at this threshold is a limitation. Fourth, the improvement in performance between the Clinical and ClinRad model is small and unlikely to change clinical management substantially. Finally, the logistic regression models employed in this analysis were insensitive to nonlinear and nonmonotonic effects.

In conclusion, we have confirmed in a prospective multicentre dataset that previously proposed GLCM features—correlation and contrast—contain incremental prognostic information. The clinicoradiomic model incorporating GLCM correlation and contrast with tumour and nodal stage, age and volume outperformed TNM stage alone in the discrimination of 3-year overall survival. Nevertheless, the level of discrimination remained modest and it is questioned if this will impact on management substantially.

## Supplementary information

Below is the link to the electronic supplementary material.

Supplementary file1 (PDF 544 KB)

## Abbreviations

AUC: Area under receiver operating characteristic curve
GLCM: Grey-level co-occurrence matrix
OS: Overall survival
TNM: Tumour-node-metastasis

## Declarations

### Guarantor

The scientific guarantor of this publication is Professor Vicky Goh.

### Statistics and biometry

One of the authors has significant statistical expertise.

No complex statistical methods were necessary for this paper.

### Study subjects or cohorts overlap

None of these study subjects or cohorts has been previously reported.

### Methodology

• prospective

• diagnostic or prognostic study

• multicentre study
