# Supplementary material for: Multicentre validation of CT grey-level co-occurrence matrix features for overall survival in primary oesophageal adenocarcinoma
Source: Eur Radiol. 2024 Mar 25;34(10):6919–28. doi: 10.1007/s00330-024-10666-y (PMC11399295; doi:10.1007/s00330-024-10666-y)
Supplement: Supplementary file 1 — Supplementary file1 (PDF 544 KB) [file 330_2024_10666_MOESM1_ESM.pdf]

SUPPLEMENTARY INFORMATION

Grey Level Co-Occurrence Matrix Feature Definitions

GLCM Correlation quantifies the linear association between adjacent voxels' grey levels

$$Correlation = \sum_{i}\sum_{j} p(i,j)\log\big(p(i,j)\big)$$

GLCM Contrast quantifies variance of adjacent voxels' grey levels

$$Contrast = \sum_{i}\sum_{j} (i-j)^2 \log\big(p(i,j)\big)$$

Here i and j denote grey level bins and $p(i,j)$ represent the co-occurrence probability of bins i and j.

Feature Variability Across Scanners and Institutions

In terms of GLCM feature variability with respect to scanner manufacturer and study institution GLCM_Correlation varied significantly with scanner manufacturer ($F_{3,161} = 14.35, P < .001$) but was stable across institutions ($F_{3,161} = 1.33, P > .05$). GLCM_Contrast was invariant to scanner manufacturer ($F_{3,161} = 1.63, P > .05$) but varied with study institution ($F_{4,160} = 2.88, P = .02$). GLCM_Correlation was stable with respect to tumour volume ($r = 0.12, P > .05$) and GLCM_Contrast was negatively confounded with respect to tumour volume ($r = -0.2, P = .01$).

SUPPLEMENTARY FIGURES

Supplementary Figure 1. Examples of tumour segmentations. Participant A: 65-year-old male with a clinically-staged T2 tumour; Participant B: 66-year-old male with a clinically-staged T2 tumour; Participant C: 56-year-old male with a clinically-staged T3 tumour; Participant D: 64-year-old male with a clinically-staged T3 tumour.

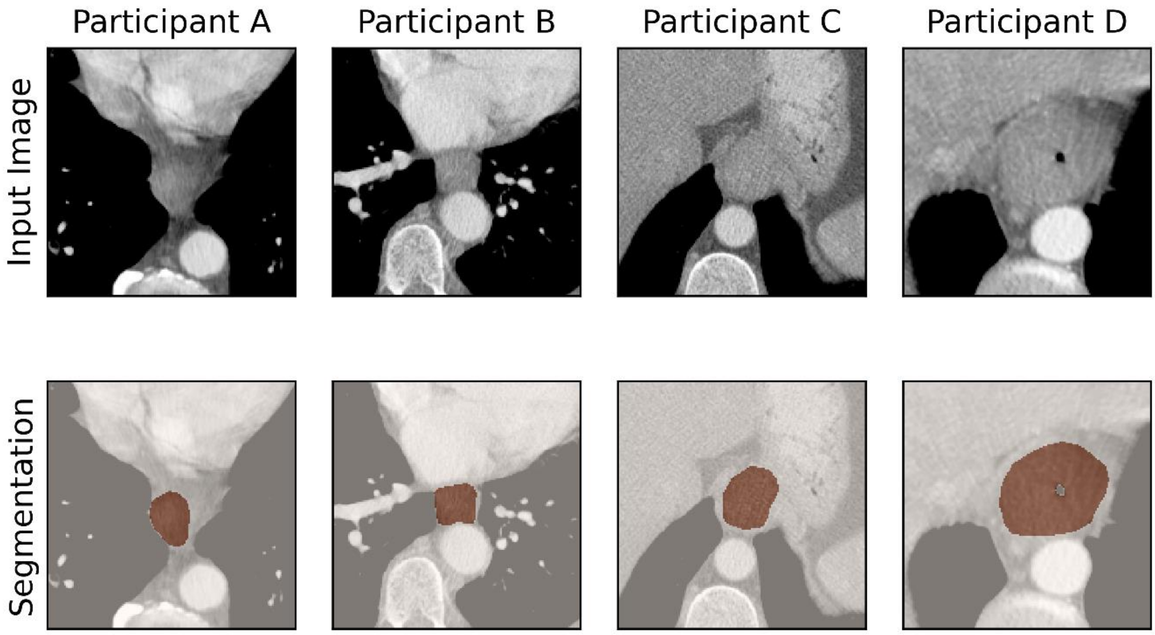

Supplementary Figure 2. Segmentation of a clinically-staged T1 tumour in a 72-year-old male.

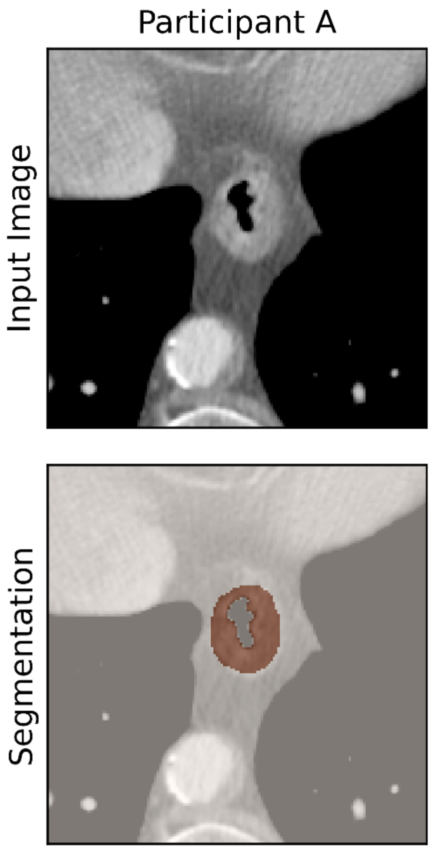

Supplementary Figure 3. Histograms of participant characteristics. cT and cN denote clinical tumour and nodal stage, as determined following consensus tumour board review. OS time denotes overall survival time in years. Treatment modalities S, CR and CRS denote surgery, chemoradiotherapy, and chemoradiotherapy and surgery, respectively.

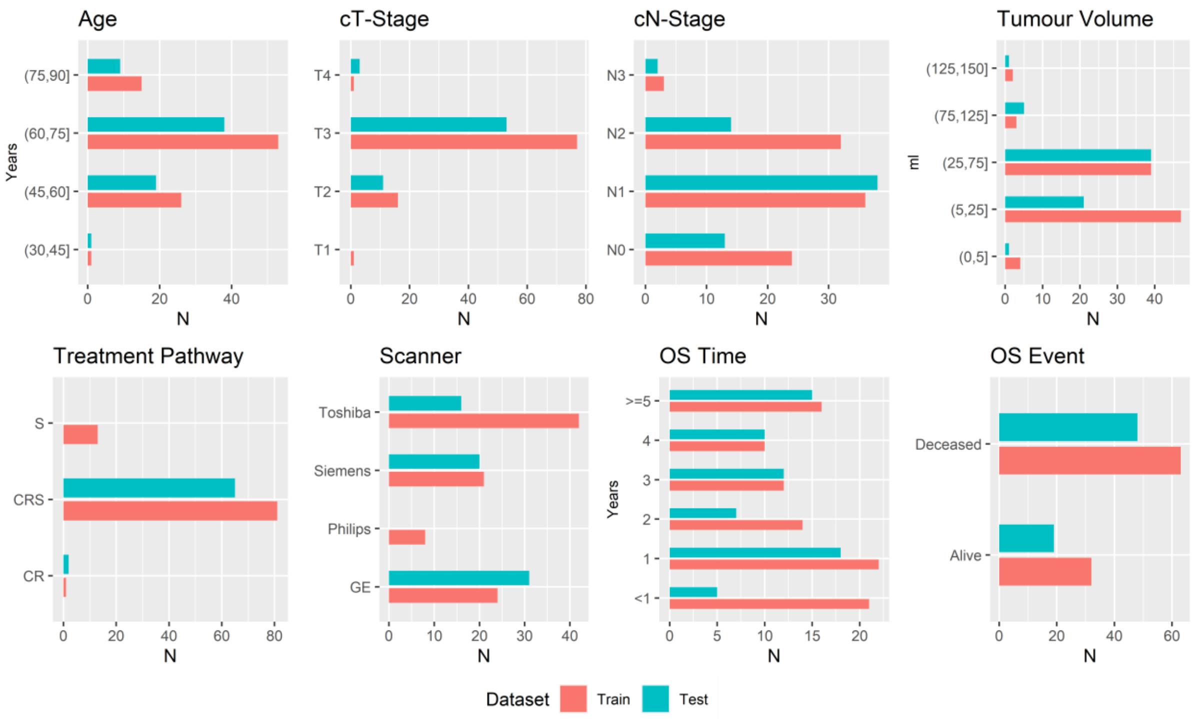

SUPPLEMENTARY TABLES

Supplementary Table 1. Image acquisition and reconstruction parameters across the datasets.

Variable	Value	Train (n)	Test (n)
Scanner Manufacturer	GE	24	31
	Philips	8	0
	Siemens	21	20
	Toshiba	42	16
Peak Voltage (kVp)	100	13	1
	120	82	52
	130	0	14
Tube Current (mA)	(0,200]	25	33
	(200,500]	53	28
	(500,1000]	3	5
	unknown	14	1
Reconstructed Voxel Size (mm)	(0.5,0.7]	13	7
	(0.7,0.9]	72	56
	(0.9,1]	10	4

Supplementary Table 2. Confirmatory model performance evaluation in per-institution testing. In each fold, four institutions were used for model development and one for testing. Area under Curve (AUC) and Brier scores were computed with respect to 3-year overall survival. Mean and standard deviation are provided.

Dataset	Model	AUC	Brier
Train	Clinical	0.66 ± 0.02	0.23 ± 0.01
Train	ClinVol	0.66 ± 0.03	0.23 ± 0.01
Train	ClinRad	0.69 ± 0.03	0.22 ± 0.01
Train	Stage	0.59 ± 0.02	0.24 ± 0.00
Test	Clinical	0.65 ± 0.11	0.23 ± 0.04
Test	ClinVol	0.63 ± 0.10	0.24 ± 0.04
Test	ClinRad	0.69 ± 0.16	0.24 ± 0.05
Test	Stage	0.62 ± 0.12	0.24 ± 0.03

Supplementary Table 3. Partial correlation analysis. Partial spearman correlations were computed over the variables age, clinical T-stage, clinical N-stage, Volume, GLCM_Correlation and GLCM_Contrast using both training and testing datasets.

Dataset	Model	Predicted Group	N	Events	Median Survival (days)	Mean Survival (days)
Train	Clinical	High Risk	82	56	932	1528 [654, 1445]
Train	Clinical	Low Risk	13	7	1563	2137 [1316, -]
Test	Clinical	High Risk	56	40	1186	1461 [793, 1579]
Test	Clinical	Low Risk	11	8	1438	1442 [1225, -]
Train	ClinVol	High Risk	77	52	932	1530 [654, 1445]
Train	ClinVol	Low Risk	18	11	1560	1982 [1252, -]
Test	ClinVol	High Risk	56	40	1187	1481 [855, 1579]
Test	ClinVol	Low Risk	11	8	1230	1318 [834, -]
Train	ClinRad	High Risk	80	55	929	1494 [654, 1295]
Train	ClinRad	Low Risk	15	8	1563	2241 [1486, -]
Test	ClinRad	High Risk	58	40	1187	1496 [855, 1579]
Test	ClinRad	Low Risk	9	8	1334	1338 [1225, -]
Train	Stage	High Risk	79	53	929	1517 [583, 1295]
Train	Stage	Low Risk	16	10	1560	2075 [1252, -]
Test	Stage	High Risk	56	40	1156	1447 [780, 1551]
Test	Stage	Low Risk	11	8	1438	1539 [1230, -]

Supplementary Table 4. Participant overall survival in each predicted risk group. N refers to the number of participants assigned to the group.

	**Age**	**T stage**	**N stage**	**Volume**	**GLCM Correlation**	**GLCM Contrast**
**T stage**	0.01 [-0.14,0.16]					
**N stage**	-0.07 [-0.22,0.11]	0.28 [0.11,0.43]				
**Volume**	-0.01 [-0.17,0.16]	0.09 [-0.07,0.24]	0.3 [0.17,0.42]			
**GLCM Correlation**	-0.01 [-0.19,0.16]	-0.1 [-0.26,0.05]	0 [-0.15,0.17]	0 [-0.19,0.18]		
**GLCM Contrast**	0.08 [-0.1,0.26]	-0.03 [-0.16,0.11]	-0.06 [-0.22,0.09]	-0.17 [-0.33,-0.01]	-0.54 [-0.66,-0.42]	
**Overall survival Time**	-0.14 [-0.3,0.02]	-0.12 [-0.26,0.04]	-0.15 [-0.33,0.02]	-0.04 [-0.2,0.13]	0.1 [-0.05,0.25]	0.02 [-0.14,0.18]
